# Supplementary material for: Bayesian inference of a spectral graph model for brain oscillations
Source: Neuroimage. Author manuscript; Available in PMC 2024 Feb 5. (PMC10840584; doi:10.1016/j.neuroimage.2023.120278)
Supplement: 1 [file NIHMS1961314-supplement-1.pdf]

Appendix. Supplementary

A.1. Spectral graph model

Notation. All the vectors and matrices are written in boldface and the scalars are written in normal font. The frequency f of a signal is specified in Hertz (Hz), and the corresponding angular frequency $\omega = 2\pi f$ is used to obtain the Fourier transforms. The connectivity matrix is defined as $\mathbf{C} = c_{jk}$, where c_{jk} is the connectivity strength between regions j and k, normalized by the row degree.

Mesoscopic model

Given region k out of N regions, we denote the local excitatory signal as $x_e(t)$, local inhibitory signal as $x_i(t)$, and the long-range macroscopic signals as $x_k(t)$. Combining the decay of individual signals, coupling of excitatory and inhibitory signals as well as input white Gaussian noise, the evolution models of $x_e(t)$ and $x_i(t)$ are:

$$\frac{\mathrm{d}x_e(t)}{\mathrm{d}t} = -\frac{f_e(t)}{\tau_e} \star \left(g_{ee}\, x_e(t) - g_{ei}\, f_i(t) \star x_i(t)\right) + p(t)\,, \text{ and,} \tag{3}$$

$$\frac{\mathrm{d}x_i(t)}{\mathrm{d}t} = -\frac{f_i(t)}{\tau_i} \star \left(g_{ii}\, x_i(t) + g_{ei}\, f_e(t) \star x_e(t)\right) + p(t)\,, \tag{4}$$

where $f_e(t)$ and $f_i(t)$ are the ensemble average neural impulse response function, $\star$ stands for convolution, $p(t)$ is input noise, parameters g_{ee}, g_{ii}, g_{ei} are neural gain terms, and parameters τ_e, τ_i are characteristic time constants, which are shared for every region k. We assume Gamma-shaped $f_e(t)$ and $f_i(t)$ as

$$f_e(t) = \frac{1}{\tau_e^2} \exp\left(\frac{-t}{\tau_e}\right) \text{ and } f_i(t) = \frac{1}{\tau_i^2} \exp\left(\frac{-t}{\tau_i}\right).$$

Macroscopic model

Accounting for long-range connections between brain regions, the macroscopic signal x_k is assumed to conform to the following evolution model:

$$\frac{\mathrm{d}x_k(t)}{\mathrm{d}t} = -\frac{1}{\tau_G} f_G(t) \star x_k(t) + \frac{\alpha}{\tau_G} f_G(t) \star \sum_{j=1}^{N} c_{jk} x_j(t - \tau_{jk}^{v}) + \left(x_e(t) + x_i(t)\right), \tag{5}$$

where, τ_G is the graph characteristic time constant, α is the global coupling constant, c_{jk} are elements of the connectivity matrix, τ_{jk}^{v} is the delay in signals reaching from the jth to the kth region, v is the cortico-cortical fiber conduction speed with which the signals are transmitted. The delay τ_{jk}^{v} is calculated as d_{jk}/v, where d_{jk} is the distance between regions j and k and $x_e(t) + x_i(t)$ is the input signal determined from Eqs. (3) and (4). The Gamma-shaped $f_G(t)$ is written as

$$f_G(t) = \frac{1}{\tau_G^2} \exp\left(\frac{-t}{\tau_G}\right).$$

The neural gain g_{ee} is kept as 1 to ensure parameter identifiability, therefore, SGM only includes 7 identifiable parameters as listed in Table 1.

Closed-form model solution in the fourier domain

A salient feature of SGM is that it provides a closed-form solution of brain oscillations under the frequency domain. Let $\mathcal{F}$ be the Fourier transform at angular frequency $\omega = 2\pi f$. Note that the mesoscopic models for different regions share the same parameters, therefore, without loss of generality, we can drop the subscript k.

The solutions for $x_e(t)$ and $x_i(t)$ under the frequency domain are

$$X_e(\omega) = \mathcal{F}(x_e(t)) = \frac{\left\{1 + \frac{g_{ei} F_e(\omega) F_i(\omega)/\tau_e}{j\omega + g_{ii} F_i(\omega)/\tau_i}\right\} P(\omega)}{j\omega + g_{ee} F_e(\omega)/\tau_e + \frac{(g_{ei} F_e(\omega) F_i(\omega))^2}{\tau_e \tau_i (j\omega + g_{ii} F_i(\omega)/\tau_i)}} = H_e(\omega) P(\omega),$$

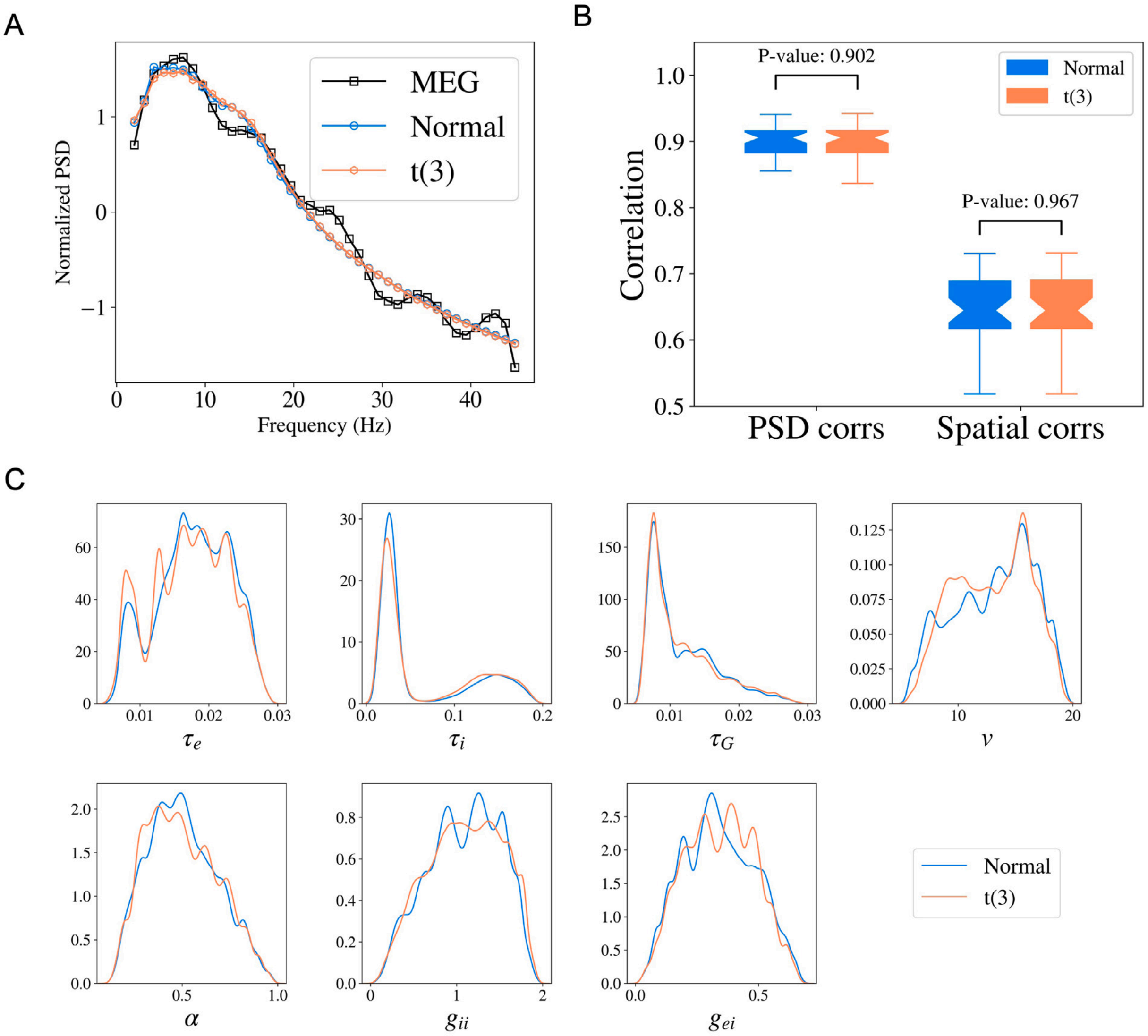

Fig. S.1. (A) The PSDs from MEG data and the SBI-SGM with normal and t(3) noises. (B) Pearson's correlation of PSD from each ROI and spatial correlation for the alpha frequency band. (C) The kernel density estimations of the posterior SGM parameters under the normal and t(3) noise.

and

$$X_i(\omega) = \mathcal{F}(x_i(t)) = \frac{\left\{1+\frac{g_{ei}F_e(\omega)F_i(\omega)/\tau_i}{j\omega+g_{ee}F_e(\omega)/\tau_e}\right\}P(\omega)}{j\omega+g_{ii}F_i(\omega)/\tau_i+\frac{(g_{ei}F_e(\omega)F_i(\omega))^2}{\tau_e\tau_i(j\omega+g_{ee}F_e(\omega)/\tau_e)}} = H_i(\omega)P(\omega),$$

where $P(\omega)$, $F_e(\omega)$, $F_i(\omega)$, $F_G(\omega)$ are the Fourier transform of $p(t)$, $f_e(t)$, $f_i(t)$, and $f_G(t)$ at angular frequency ω.

We define the complex Laplacian matrix $\mathcal{L}(\omega) = \mathbf{I} - \alpha\mathbf{C}^*(\omega)$ where $\mathbf{C}^*(\omega) = [c_{ij}\exp(-j\omega\tau_{ij}^{v})]_{i,j=1,\dots,N}$. The solution of the macroscopic signals at a angular frequency ω is

$$\mathbf{X}(\omega) = [\,\mathcal{F}(x_1(t)),\dots,\mathcal{F}(x_N(t))]^{\mathrm{T}} = \left(j\omega + \frac{1}{\tau_{\mathbf{G}}}F_{\mathbf{G}}(\omega)\mathcal{L}(\omega)\right)^{-1} H_{\mathrm{local}}(\omega)\mathbf{P}(\omega), \tag{6}$$

where $H_{\mathrm{local}}(\omega) = H_e(\omega) + H_i(\omega)$.

From here, we can re-write $\mathbf{X}(\omega)$ by using the eigendecomposition of the complex Laplacian matrix $\mathcal{L}(\omega)$ which is:

$$\boldsymbol{\mathcal{L}}(\omega) = \boldsymbol{U}(\omega)\boldsymbol{\Lambda}(\omega)\boldsymbol{U}(\omega)^H, \tag{7}$$

where, $\boldsymbol{U}(\omega)$ are the eigenvectors and $\boldsymbol{\Lambda}(\omega) = \mathrm{diag}([\lambda_1(\omega),\dots,\lambda_N(\omega)])$ consist of the eigenvalues $\lambda_1(\omega),\dots,\lambda_N(\omega)$, at angular frequency ω.

By using the above eigen-decomposition of the Laplacian matrix, the $\mathbf{X}(\omega)$ can be re-written as:

$$\mathbf{X}(\omega) = \sum_{k=1}^{N} \frac{\boldsymbol{u}_k(\omega)\boldsymbol{u}_k(\omega)^{\mathrm{H}}}{j\omega + \tau_G^{-1}\lambda_k(\omega)F_G(\omega)} H_{\mathrm{local}}(\omega)\mathbf{P}(\omega), \tag{8}$$

where, $\boldsymbol{u}_k(\omega)$ are the eigenvectors from $\boldsymbol{U}(\omega)$ and $\lambda_k(\omega)$ are the eigenvalues from $\boldsymbol{\Lambda}(\omega)$ obtained by the eigen-decomposition of the Laplacian matrix $\boldsymbol{\mathcal{L}}(\omega)$ obtained in Eq. (7). Eq. (8) is the closed-form steady state solution of the macroscopic signals at a specific angular frequency ω. As SGM provides a closed-form solution $\mathbf{X}(\omega)$, we can compare the modeled and empirical power spectra to estimate the global parameters.

A.2. SBI-SGM under the heavy-tailed noise

To investigate the performance of SBI-SGM under the heavy-tailed noise, we conduct the numerical study with the noise from Student's t distribution with degree of freedom 3. We further control the standard deviation of the noise at 1.6 to compare the results with noise $N(0, 1.6^2)$ in Section 3.4, i.e., the density function of our heavy-tailed noise is $f(x) \propto (1+x^2/2.56)^{-2}$. Except for the random noise, all the other settings are identical to the setting in Section 3.4 for the comparison.

In the 10 repetitions with t(3) noise, the PSD Pearson's correlation between the reconstructed and empirical PSDs is changed in [0.9049, 0.9066] which is very similar to the results with the Gaussian noise ([0.905, 0.907] in Section 3.4). We also present the results from the representative experiment that yields a correlation closest to the mean level in the 10 repetitions for both noise types in Fig. S.1, including the reconstructed PSD, the PSD and spatial (in alpha band) correlations and the posterior densities of the 7 SGM parameters. All the results are very similar under both noise types.

The comparison between the two error types indicates that the SBI-SGM is robust to the selection of the noise distribution.
